# Dense PVDF-type polymer-in-ceramic electrolytes for solid state lithium batteries†

**DOI:** 10.1039/d0ra03433a

**Published:** 2020-06-11

**Authors:** Jiajie Wu, Xiaomeng Wu, Wenli Wang, Qian Wang, Xiaoyu Zhou, Yang Liu, Bingkun Guo

**Affiliations:** Materials Genome Institute, Shanghai University Shanghai 200444 China liuyang81@shu.edu.cn guobingkun@shu.edu.cn; Space Power Technology State Key Laboratory, Shanghai Institute of Space Power-Sources Shanghai 200245 P. R. China; Guangdong Provincial Key Laboratory of Energy Materials for Electric Power, Southern University of Science and Technology Shenzhen 518055 China

## Abstract

Li_7_La_3_Zr_1.4_Ta_0.6_O_12_ (LLZTO) and polyvinylidene fluoride (PVDF) composite electrolytes (LPCEs) with a high ceramic content up to 80 wt% have been developed. Hot pressing can significantly reduce the porosity of LPCEs and increase the conductivity to 1.08 × 10^−4^ S cm^−1^ at 60 °C, then the LPCEs can sustain Li plating/stripping cycling for over 1500 h, and make LiFePO_4_/LPCE/Li cell display a capacity retention of 86% in 200 cycles.

## Abstract 2

Li_7_La_3_Zr_1.4_Ta_0.6_O_12_ (LLZTO) and polyvinylidene fluoride (PVDF) composite electrolytes (LPCEs) with a high ceramic content up to 80 wt% have been developed.

## Graphical abstract

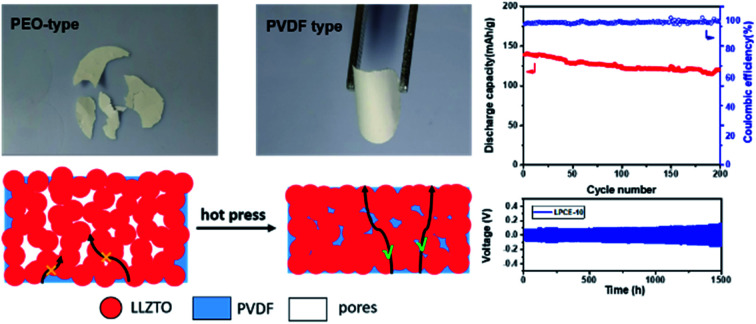

## Introduction

Driven by ever-increasing demand on high-energy-density batteries in the field of electric vehicles and portable electronics, high-capacity cathodes and anodes, such as Ni-rich cathodes and Si-based anodes, were rapidly adopted into the battery industry.^1,2^ Meanwhile, safety problems have been appeared due to the highly active electrodes and flammable liquid organic electrolytes. A breakthrough in battery technology is urgent to meet the requirements on high energy density and safety.^3,4^ Unlike liquid organic electrolytes, solid-state electrolytes (SSEs) have nonflammability, good thermal stability, and high electrochemical stability.^5,6^ Moreover, SSEs can present excellent compatibility with lithium metal anodes. Therefore, solid-state batteries (SSBs) are considered to have great potential to increase energy density and eliminate the safety issues simultaneously.^7,8^

Various solid electrolytes, including inorganic solid electrolytes (ISEs) and solid polymer electrolytes (SPEs), have distinctive advantages and disadvantages. Garnets, as one of the most promising inorganic solid electrolytes, have high chemical stability, high ionic conductive of ∼10^−3^ mS cm^−1^, and high stability against to lithium metal anodes and high voltage cathodes. However, the critical problems related to garnet ceramic electrolytes, such as the poor interfacial contact with electrodes and the fragility of garnet ceramic thin film, limit their application in SSBs.^9–12^ Compared to inorganic electrolytes, polymer electrolytes are highly flexible and compatible with electrodes. However, the low ionic conductivity is a big challenge for the practical applications of polymer electrolytes. Even though various modification methods have been studied such as polymer bending,^13^ cross-linking,^14^ adding ionic liquids or plasticizers,^15^ and synthesis inorganic or metal–organic frameworks fillers.^16,17^ The combination of ceramic particles with polymer electrolytes to form composite electrolytes has been proved to be an effective strategy for designing high-performance solid electrolytes.

Polymer/ceramic composite electrolytes can be briefly divided into two categories: “ceramic-in-polymer” (CIP, low ratio of ceramic) and “polymer-in-ceramic” (PIC, high ratio of ceramic).^18^ For the CIP electrolytes, the ceramic particles not only increase the ionic conductivity of electrolytes, but also enhance their electrochemical stability.^19^ Recently, various affecting factors of the CIP electrolytes, such as the type of fillers,^20^ particle size of fillers,^21^ and lithium salt content,^22^ have been investigated. The ionic conductivity of CIP electrolytes has achieved to 10^−4^ S cm^−1^, which can make solid-state cells operate at room temperature.^23^ Even though there is great progress in improving ionic conductivity after continuous optimization, the Li dendrites can hardly be suppressed by this type of electrolytes. Compared to CIP electrolytes, PIC electrolytes have been demonstrated to be more effective on suppressing lithium penetration and improving safety.^24^ However, high ratio of ceramic leads to the poor flexibility and low ionic conductivity of PIC electrolytes, especially for the typical PEO/garnet composite electrolytes. For example, high content LLZTO particles/PEOs composite electrolytes with PEG as binder obtained by a hot pressing provide high ionic conductivity more than 10^−4^ S cm^−1^ at 55 °C and can effectively suppress Li dendrites.^25^ However, without the binder of PEG, cracks could be easily found in PEO-type PICs and lead the PICs hard to be applied in SSBs. Unlike PEO-type PICs, PVDF-type PICs can bond ceramic fillers better and address a dense electrolyte membrane by the hot pressing method. Moreover, PVDF has the improved electrochemical, thermal, and mechanical stability compared to PEO.^23^

In this work, we developed a PIC electrolyte, LLZTO and PVDF composite electrolyte (LPCE). Compared to PEO, PVDF possesses stronger adhesivity, which ensures the high flexibility of LPCE even with a content of LLZTO up to 80 wt%. The influence of hot pressing on the conductivity of LPCEs were also investigated. Hot pressing can significantly reduce the porosity of LPCEs and increase the conductivity to 1.08 × 10^−4^ S cm^−1^ at 60 °C. The LPCE membranes show excellent Li dendrite suppression property and sustain Li plating/stripping cycling for over 1500 h. Moreover, the solid LiFePO_4_/LPCE/Li cells displayed long cycling stability with a capacity retention of 86% in 200 cycles.

## Results and discussion

**Fig. 1:**
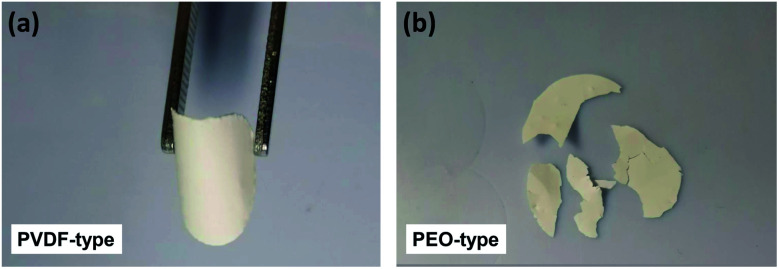
Photographs of the PVDF-type PIC membrane (a) and the PEO-type PIC membrane (b).

For comparison, Fig. 1a and b show the pictures of the PVDF-type PIC membrane and PEO-type PIC membrane with 10 wt% polymer and 80 wt% LLZTO. It can be seen that the PVDF-type PIC membrane (Fig. 1a) exhibits great flexibility and no crack can be observed even after repeated bending, which could be ascribed to the strong adhesivity and high mechanical strength of PVDF. By contrast, the PEO-type PCI membrane displays poor flexibility and easily tend to chips (Fig. 1b).

**Fig. 2:**
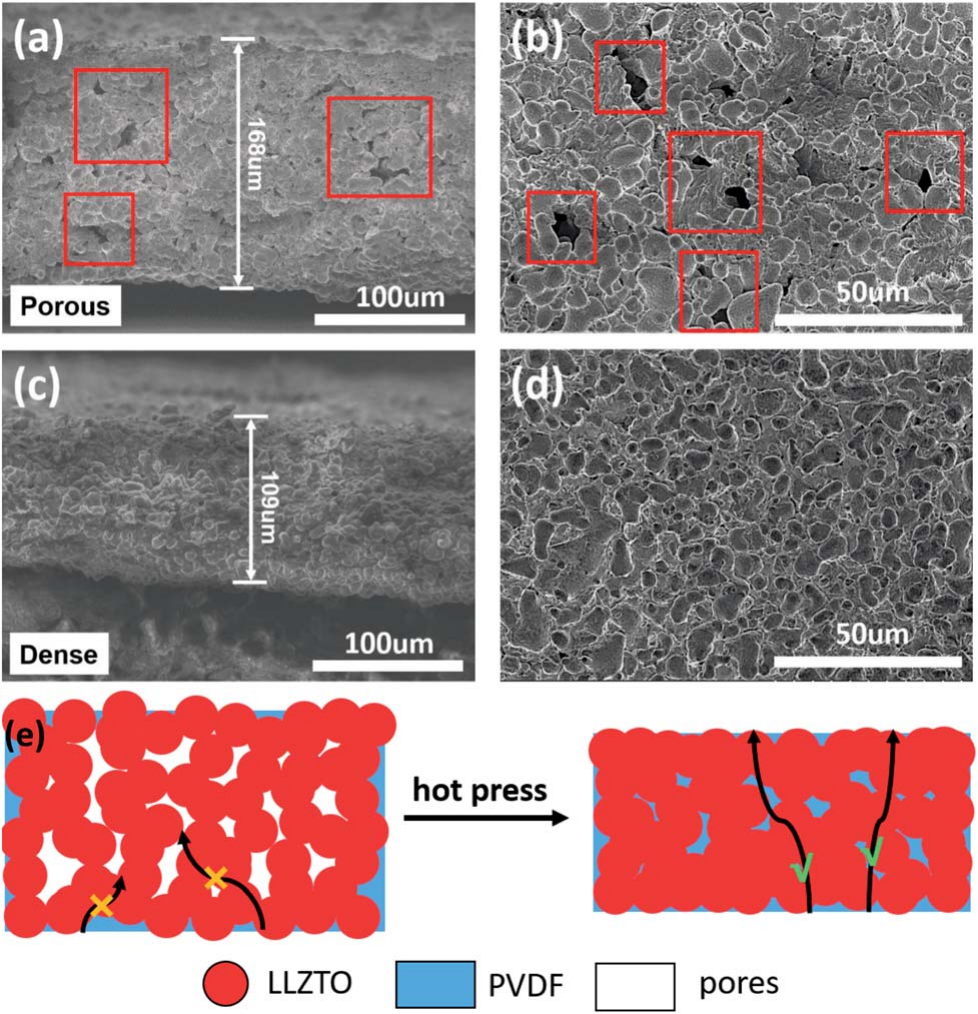
The cross-sectional (a) and top-view (b) SEM images of LPCE without hot pressing. The cross-sectional (c) and top-view (d) SEM images of LPCE hot pressing at 20 MPa. The schematic illustrations of density influence the Li^+^ transport in high fillers content solid electrolytes (e).

To elucidate the morphology and density of LPCE, the SEM images for the LPCE with 10 wt% LiTFSI (marked as LPCE-10% LiTFSI) before and after hot pressing are shown in Fig. 2. As shown in Fig. 2a and b, LPCE without hot pressing has some pores with typical pore size of 3–7 μm, which could be resulted by solvent evaporation. The porous structure could impede Li^+^ transport between PVDF and LLZTO particles, leading to the poor ionic conductivity. It is obvious that the LPCE become dense after hot pressing at 20 MPa (Fig. 2c and d), which will benefit Li^+^ transport in the electrolyte. As shown in Fig. 2c and d, the typical film thickness of LPCE after hot pressing is ∼100 μm and LLZTO particles are embedded in the PVDF matrix homogeneously. Moreover, the LPCE membrane after hot pressing at 20 MPa has smoother surfaces, which can provide better contact with electrodes. Fig. 2e schematic illustrates that even though PVDF can bend LLZTO particles tightly, the LPCE membrane still has a lot of pores left by solvent evaporation. Hot pressing the as-prepared LPCE membrane could address dense structure which provides continuous Li^+^ pathways and remarkable ionic conductivity. The XRD patterns of as-synthesized LPCE and LLZTO powders are shown in Fig. S1a,† respectively. The peaks of LLZTO powders are well marched to a cubic-phase garnet structure (Joint Committee on Powder Diffraction Standards card #80-0457). No significant change was found within the as-prepared LPCE membranes, implying good stability of the LLZTO ceramic with PVDF in the preparation process. Thermogravimetric analysis (TGA) was conducted to evaluate the thermal stability of the electrolytes as shown in Fig. S1b.† It can be seen that the LPCE is stable until 340 °C and the mass loss at 350–450 °C is considered as the decomposition of PVDF and LiTFSI. Compared to the organic liquid electrolytes, the LPCE shows much better thermal stability.

**Fig. 3:**
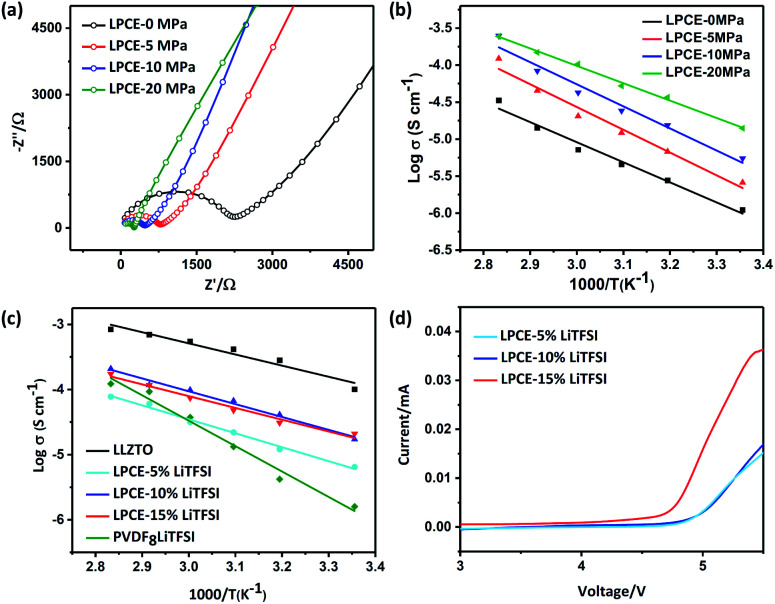
The impedance plots (a) and Arrhenius plots of conductivity (b) for LPCEs after hot pressing at different pressure. (c) Arrhenius plots of conductivity for LPCEs with various LiTFSI contents, compared to a LLZTO pellet and the PVDF_8_LiTFSI SPE. (d) The LSV plot with various LiTFSI contents at the scan rate of 0.5 mV s^−1^.

To confirm our assumption that the membrane which polymer and ceramic particles has good contact can enhance ionic conductivity, electrochemical impedance spectroscopy (EIS) measurements of LPCE membranes before and after hot pressing were carried out. All of the Nyquist plots of LPCE hot pressing at different pressure at 60 °C summarized in Fig. 3a. The results show well fit into two parts: a suppressed semicircle at the middle–high frequency and a linear part at a low frequency. The semicircle is associated with the bulk electrolyte resistance and the linear part is corresponding to the capacitive behavior of Li-ion-blocking electrode.^22,26^ The ionic conductivity of LPCEs calculated from the Nyquist plot increases with increasing hot-pressing pressure, the ionic conductivity of LPCEs without hot pressing is 7.17 × 10^−6^ S cm^−1^, whereas the membrane hot pressed at 20 MPa is 1.08 × 10^−4^ S cm^−1^, which is hundreds times higher than that of the sample without hot pressing. The conductivities of LAPEs among 25 to 80 °C are shown in Fig. 3b. When increasing the temperature of the different electrolyte membranes, the conductivities of the LPCEs gradually increase linearly, showing thermally activated conduction. All curves are consistent with the Arrhenius plots.^27^ The LPCE hot pressed at 20 MPa shows the lowest activation energy of 0.39 eV, which is close to the activation energy of the LLZTO pellet.^9^ Clearly, hot pressing can make the better contact of polymer-particle and help to build continuous pathways between LLZTO particles and PVDF, which enhance the ionic conductivity of LPCE.

In addition, the LiTFSI concentrations in the LPCEs were varied from 5 to 15 wt% (marked as LPCE-5, LPCE-10, LPCE-15, respectively) to investigate the influence of lithium salt concentration. It is obvious that conductivity of LPCE-10 and LPCE-15 are very close and much higher than that of the LPCE-5, indicating 10% of LiTFSI is enough for the LPCEs. Moreover, the conductivity of PVDF_8_LiTFSI SPE is about 3.74 × 10^−5^ S cm^−1^ at 60 °C, much lower than that of LPCE-10, indicating that the compositing of PVDF and LLZTO can sufficiently improve the SSE's ionic conductivity.

Electrolytes with large electrochemical window can be used for high voltage cathode, which can increase the energy density of the lithium-ion battery.^21^ The electrochemical stabilities of LPCEs with different concentration of LiTFSI were evaluated by cycle voltammetry at 60 °C as shown in Fig. 3d. Both of LPCE-10 and LPCE-5 exhibit the high oxidative stabilities up to 4.7 V *vs.* Li^+^/Li. In summary, LPCE-10 has both high ionic conductivity and good electrochemical stability.

**Fig. 4:**
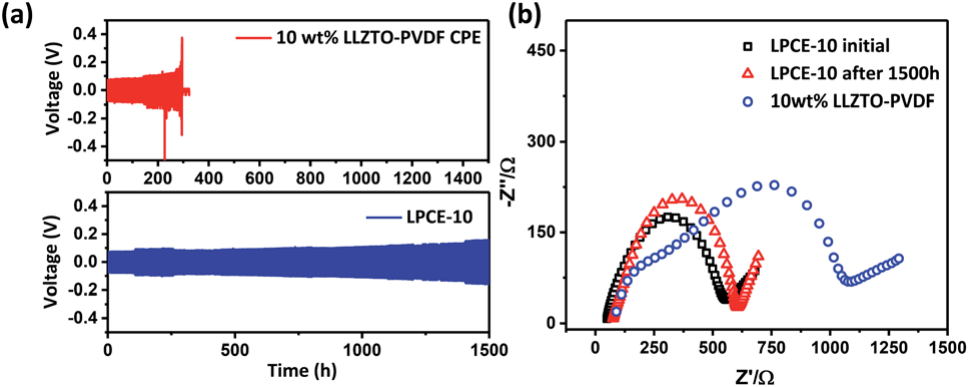
(a) Plating and striping cycling test of Li|LPCE-10|Li and of Li|10 wt% LLZTO–PVDF CPE|Li cells under current density of 0.1 mA cm^−2^ at 60 °C. (b) The impedance plots of symmetric cell of LPCE-10 and 10 wt% LLZTP-PVDF CPE.

The stability of the electrode–electrolyte interface is a critical factor to influence the cycling performance.^26^ To verify the Li dendrite suppression property of LPCEs, lithium symmetric cells using LPCE-10 and 10 wt% LLZTO–PVDF composite electrolyte which reported as the best ratio at the low ceramic content PVDF-type composite electrolytes were tasted under current density of 0.1 mA cm^−2^ at 60 °C.^23^ As shown in Fig. 4a, the cell using LPCE-10 tested in the same conditions shows low polarized voltage of 0.09 V and stable cycle performance over 1500 h, whereas the 10 wt% LLZTO–PVDF composite electrolyte reaches a short circuit within 298 h. Clearly, the LPCE-10 with high ceramic content is more effective on suppression of lithium penetration. Moreover, the impedance of Li|LPCE-10|Li cell shown in Fig. 4b illustrates only a small increase of 32 Ω after 1500 h cycling, suggesting that the LPCE membrane is electrochemical stable against lithium metal. Although LPCE-10 presents lower ionic conductivity than pure LLZTO (Fig. 3c), the impedance of LiFePO_4_|LPCE-10|Li operated at 60 °C is 209.6 Ω (Fig. S3a†), which is much lower than that of LLZTO.^30^ This should be related to the flexibility of LPCE-10 as shown in Fig. 1a.

The SEM images shown in Fig. S2† exhibit the morphology of Li anode after cycling as shown in Fig. 4a. The anode using LPCE-10 has a smooth surface, whereas the anode surface using 10 wt% LLZTO–PVDF composite electrolyte is uneven, implying that LPCE-10 can effectively inhibit dendrite growth.

**Fig. 5:**
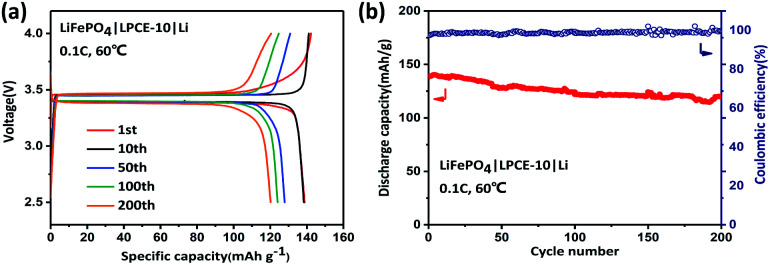
Typical charge–discharge curves (a) and cycle performance (b) of LiFePO_4_|LPCE-10|Li battery at 0.1 C. All tested batteries were operated at 60 °C.

To evaluate the electrochemical performance of the LPCE, Li|LPCE-10LiTFSI|LiFePO_4_ cells were investigated at 60 °C in the voltage range from 2.5 to 4.0 V *vs.* Li^+^/Li at 0.1 C. Fig. 5a and b show the typical charge–discharge curves and cycle performance. The LPCE cell displays a high reversible capacity of 141 mA h g^−1^ with a coulombic efficiency of 98.5% (Fig. 5a). The charge–discharge curves are as flat as that in liquid electrolyte cells and the polarization is also very small, indicating the low interfacial impedance of the solid state cell. Furthermore, no obvious increase of the polarization can be observed even after 200 cycles, suggesting that LPCE have high stability against to both of lithium metal and cathode. As shown in Fig. 5b, the LPCE cell reveals good cycling stability with a high capacity retention of 86% even after 200 cycles. The cell also achieves a good rate performance (Fig. S3b†) with capacities of 136.4, 131.9, 122, 102, and 67.3 mA h g^−1^ at current rates of 0.1, 0.2, 0.5, 0.8 and 1 C, respectively. Compared with the works reported recently in Table S1,† LPCE-10 shows relatively high ionic conductivity and reversible capacity of the cell at 60 °C.^28–33^ Our work also shows a relatively simple way for cell preparation.^30^ These suggest the strategy of SSE preparation shown in this work would be promising for the application of high energy lithium batteries.

## Conclusions

In summary, LLZTO/PVDF composite electrolytes with a high ceramic content up to 80 wt% have been successfully fabricated. The composite electrolytes exhibit good flexibility due to the strong adhesivity and high strength of PVDF. Hot pressing can significantly reduce the porosity of LPCEs and increase the conductivity to 1.08 × 10^−4^ S cm^−1^ at 60 °C. The LPCE membranes show excellent Li dendrite suppression property and sustain Li plating/stripping cycling for over 1500 h. Benefiting from LPCE's low interfacial impedance and high stability against to both of lithium metal and cathode, the solid LiFePO_4_/LPCE/Li cells display low polarization and long cycling stability with a capacity retention of 86% even after 200 cycles. All these results show that the LPCE has great potential to be used for the electrolyte in solid-state lithium batteries.

## Conflicts of interest

There are no conflicts to declare.

## Supplementary Material

RA-010-D0RA03433A-s001
