# Supplementary material for: Dense PVDF-type polymer-in-ceramic electrolytes for solid state lithium batteries
Source: RSC Adv. 2020 Jun 11;10(38):22417–21. doi: 10.1039/d0ra03433a (PMC9054578; doi:10.1039/d0ra03433a)

Electronic Supplementary Material (ESI) for RSC Advances.
This journal is © The Royal Society of Chemistry 2020

**Supporting Information**

**Dense PVDF-type polymer-in-ceramic electrolytes for solid state lithium batteries**

Jiajie Wu,[a] Xiaomeng Wu,[b] Wenli Wang,[a] Qian Wang,[a] Xiaoyu Zhou,[a] Yang Liu,*[a, c]

Bingkun Guo*[a]

[a] Materials Genome Institute, Shanghai University, Shanghai 200444, China

1. Experimental section.
2. Tab.S1. Comparison of the SSEs in this and other reported work.
3. Fig.S1 XRD pattern (a) and TGA curves(b)of LLZTO powders and LPCE with different content of LiTFSI.
4. Fig.S2 SEM images of Li metal anode with LPCE-10(a) and 10 wt.% LLZTO-PVDF CPE (b).
5. Fig.S3 EIS plot(a) and rate capacity(b) of $LiFePO_4$|LPCE-10|Li operated at 60 ℃.

1. Experimental section

*Materials Synthesis.*

$Li_{6.4}La_3Zr_{1.75}Ta_{0.6}O_{12}$ particles were purchased from Milliren Technologies, Inc. and stored under argon atmosphere to prevent further aging. Poly(vinylidene fluoride) (PVDF, Mw = ~10000) , LiTFSI and other reagents were obtained from Sigma with a purity > 99% (battery grade) was dried for 24 h at 110 ℃ under vacuum.

The components of the investigated electrolytes were mixed in the binary solvent according toa stoichiometric ratio of LiTFSI: PVDF: LLZTO varies from 0.5:1:8 to 1.5:1:8. Then the mixture stirring at 60 ℃ for 6 h. The homogenized mixture was casted on a PTFE plate. The casted electrolytes were dried in a vacuum oven at 70 ℃ for 24 h to remove the trace solvent. The free-standing LPCE membranes were hot pressed for 5 min under 0, 5, 10 and 20 MPa to obtain different LPCE membranes.

*Characterizations Methods*

Electrochemical impedance spectroscopy (EIS) was measured on a high performance frequency analyzer (Novocontrol) with temperatures varied from 25 to 80 ℃. The EIS frequency was set from $10^7$ to $10^{-1}$ Hz with a perturbation voltage of 10 mV. Tests were performed by symmetrical blocking cells with the LPCE membrane inserted between two stainless steel (SS) electrodes. Cycle voltammetry (CV) was measured by SS/LPCE/Li cells on an electrochemical workstation (Solartron, FRA 1455A), which was conducted from 2 to 6 V at a scan rate of 50 mV $S^{-1}$. A field emission scanning electron microscopy (SEM, Hitachi SU8230) was used to examine the top view and cross-section morphology of the different LPCEs and Li metal anode after cycling. X-ray diffraction (XRD) of LLZTO particles and composite electrolytes were performed by D2 (BRUKER, Germany), and the operation condition was set up at 30 kV and 10 mA. The testing range was from 10 to 90° at 2° $min^{-1}$ with 0.02° step size.

The dynamic interfacial stability of the electrolyte/lithium electrode was evaluated

by a lithium-symmetrical cell via lithium deposition/stripping test, which was conducted by galvanostatically charging for 1 h and discharging for 1 h at the current density of 0.1 mA $cm^{-2}$. The cells' properties were tested by $LiFePO_4$/LPCE/Li cells on a LAND cell test system. The $LiFePO_4$, SurperP, PVDF with an optimal weight ratio of 8:1:1:1 was mixed in N-Methyl pyrrolidone(NMP). The slurry was coated on an aluminum foil and dried at 70 ℃ in a vacuum oven for 18h. The obtained $LiFePO_4$ cathode was infiltrated with a homogeneous polymer electrolyte solution (PVDF/LiTFSI/NMP) and subsequently dried at 80℃ to form the composite cathode. The mass loading of the active material is around 2 mg $cm^{-2}$. The cycling and rate performances were tested under galvanostatic conditions between 2.5 to 4 V vs. $Li^+$/Li at various current densities.

2. Tab.S1. Electrochemical performances of the SSE in this work vs. other reported SSEs.

| SSEs | Conductivity / S $cm^{-1}$ | Discharge capacity (LFP/Li cell with SSBs) | Ref. |
|---|---|---|---|
| 70 wt.% LGPS-17.4 wt.%PEO-12.6 wt.%LiTFSI | $2.2 \times 10^{-4}$(30℃) | / | 29 |
| 62.5 wt.%LGPS-15.6 wt.%PEO-21.9 wt.%LiI | $1.1 \times 10^{-5}$(60℃) | / | 30 |
| LLZTO-PEO asymmetric solid electrolyte | $1.13 \times 10^{-4}$(60℃) | 155.1 mA h $g^{-1}$ at 60℃ | 31 |
| 80 wt.% LLZTO-12.1 wt.%PEO-7.9 wt.%LiTFSI | $3.2 \times 10^{-5}$(30℃) | 118.6 mAh $g^{-1}$ at 60℃ | 32 |
| 70 wt.% LATP-30 wt.%PEO | $4 \times 10^{-5}$(25℃) | / | 33 |
| 60 wt.% LATP-28 wt.%PEO-12 wt.%LiTFSI | $1.6 \times 10^{-5}$(20℃) | 166 mAh $g^{-1}$ at 80℃ | 34 |
| 80 wt.% LLZTO-10 wt.%PEO-10 wt.%LiTFSI | $1.08 \times 10^{-4}$(60℃) | 141 mAh $g^{-1}$ at 60℃ | This work |

3. Fig.S1 XRD pattern (a) and TGA curves(b)of LLZTO powders and LPCE with different content LiTFSI.

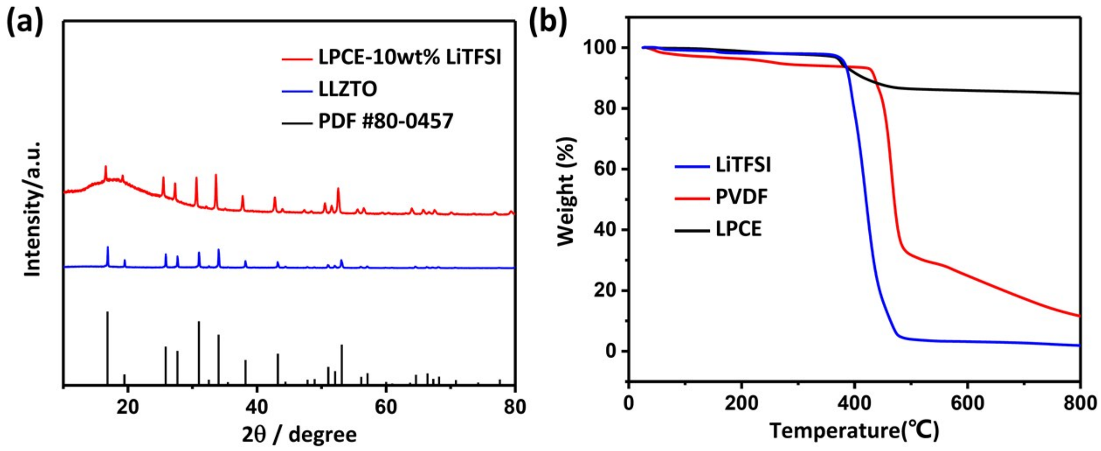

4. Fig.S2 SEM images of Li metal anode with LPCE-10 (a) and with 10 wt % LLZTO-PVDF CPE (b).

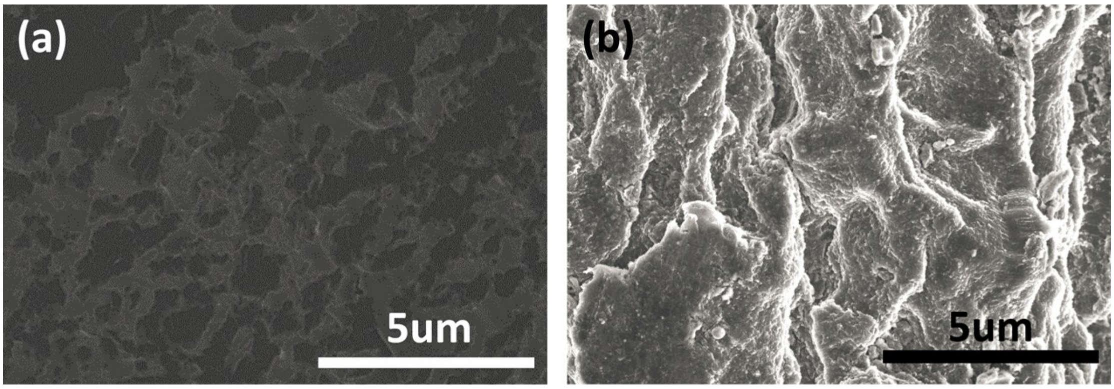

5. Fig.S3 EIS plot(a) and rate capacity(b) of $LiFePO_4$|LPCE-10|Li operated at 60 ℃.

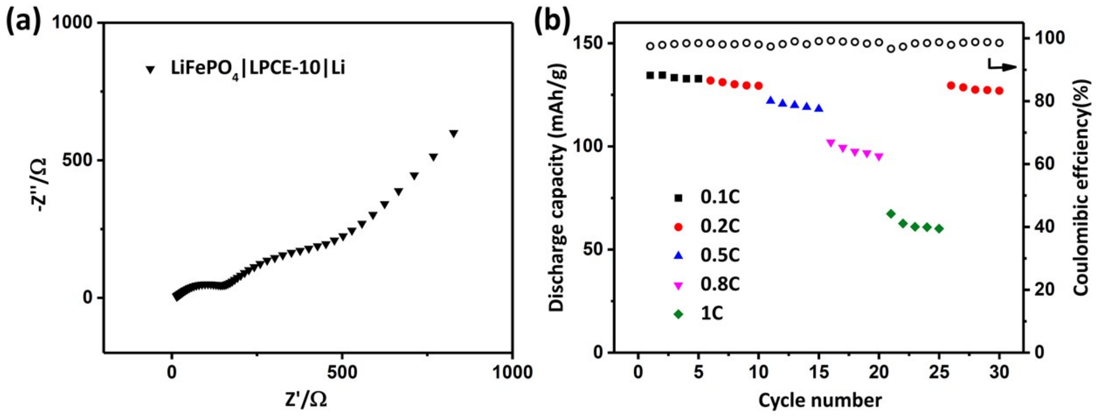

Supplement: RA-010-D0RA03433A-s001 [file RA-010-D0RA03433A-s001.pdf]
